# Intercostal Nerve to Long Thoracic Nerve Transfer for the Treatment of Winged Scapula: A Cadaveric Feasibility Study

**DOI:** 10.7759/cureus.1898

**Published:** 2017-11-30

**Authors:** Robert G Louis Jr, Joshua D Whitesides, Theofanis F Kollias, Joe Iwanaga, R. Shane Tubbs, Marios Loukas

**Affiliations:** 1 Department of Anatomical Sciences, St. George's University School of Medicine, Grenada, West Indies; 2 Department of Anatomical Sciences, St. George's University School of Medicine, Grenada, West Indies; 3 Seattle Science Foundation; 4 Neurosurgery, Seattle Science Foundation

**Keywords:** long thoracic nerve, winged scapula, intercostal nerve, nerve transfer, neurotization

## Abstract

There are very few surgical options available for treating a patient with winged scapula caused by a long thoracic nerve (LTN) injury. Therefore, we devised a novel technique based on a cadaveric dissection whereby regional intercostal nerves (ICN) were harvested and transposed to the adjacent LTN in 10 embalmed cadavers (20 sides). The LTN was identified along the lateral border of the serratus anterior and ICNs were identified at the mid-axillary line inferior to the lower edge of the pectoralis major muscle. Along the mid-clavicular line, each ICN was transected and transposed to the adjacent LTN. The length and diameter of each ICN available for mobilization to the LTN were measured. All measurements were made with microcalipers. Within the operative site, the mean proximal and distal diameters of the LTN were 1.6 and 1.1 mm, respectively. The adjacent ICN had a mean diameter of 1.3 mm. On all sides, the ICN branches were easily transposed to the adjacent LTN without any tension. Anastomosis to the LTN was performed to the third through sixth ICN provided each intercostal was preserved and mobilized anteriorly at least as far as the midclavicular line. The end to end size match between donor and LTN was appropriate on all sides. We found that it is feasible to harvest adjacent ICNs and move these to the adjacent LTN. Such a procedure, after being confirmed in patients, might offer a new technique for restoring protraction following an LTN injury.

## Introduction

Modern surgical options for the treatment of brachial plexus injuries include nerve grafting of viable nerve roots, nerve transfers, free-functioning muscle transfers, tendon transfers and combinations of these techniques [1]. The long thoracic nerve (LTN) is most commonly formed by the roots of C5 to C7. After piercing the middle scalene muscle, the upper two roots (C5-C6) join the lower root (C7) dorsal to the brachial plexus and descend posterior to the first segment of the axillary artery to reach the serratus anterior muscle [2]. Travelling along the lateral surface of the serratus anterior muscle, adherent to the chest wall, the LTN supplies branches to each of its digitations. The serratus anterior muscle is one of the most powerful muscles of the pectoral girdle, acting as a strong protractor of the scapula such as when reaching or punching. It binds the scapula to the lateral chest wall and thus enables other muscles to use it as a fixed site for movement of the upper limb [3]. The LTN is relatively unique among motor nerves in that it travels on the superficial surface of the muscle, which it innervates. It is this superficial course that exposes the LTN to injury through a variety of mechanisms. Iatrogenic LTN injury has been reported following numerous surgical procedures including thoracic and cardiac surgeries, mastectomy, axillary dissection, breast augmentation, chest tube placement, rib resection, infraclavicular anesthesia, sympathectomy, internal jugular vein cannulation, and surgical positioning [4-14]. The LTN is also subject to injury during both occupational and recreational activities in which the shoulder girdle is placed under heavy loads [6-8, 10]. According to Tubbs, et al., other procedures that might injure the LTN include ventriculopleural shunt placement, pocket dissection to implant batteries for vagal nerve stimulators, and dissecting intercostal nerves (ICN) for nerve transfer procedures [13]. The LTN (along with the suprascapular nerve) is also the most common nerve to be affected by both hereditary and idiopathic neuralgic amyotrophy (e.g., Parsonage-Turner syndrome) [15].

When the serratus anterior muscle is paralyzed as a result of LTN injury, the medial border of the scapula moves laterally and posteriorly, away from the thoracic wall, giving the appearance of a wing (scapula alata). This is exaggerated by resistance to protraction, as in pushing or leaning against a wall [16]. When the arm is raised, the upper limb may not be able to be abducted above the horizontal position as the serratus anterior muscle is unable to rotate the glenoid fossa superiorly to allow complete abduction of the arm [3]. Although relatively common, few surgical options exist for treating patients with a winged scapula. Therefore, the current cadaveric feasibility study was performed to elucidate a potential and additional surgical procedure for treating this debilitating ailment.

## Materials and methods

To test the surgical feasibility of ICN to LTN neurotization, 10 embalmed human cadavers (20 sides) were studied. The specimens consisted of males (four) and females (six) with a mean age of 73 (range 59–89 years) at the time of death. None of the cadavers exhibited any evidence of gross disease, previous surgical procedures, or traumatic lesions to the brachial plexus, axilla or chest wall.

**Figure 1:**
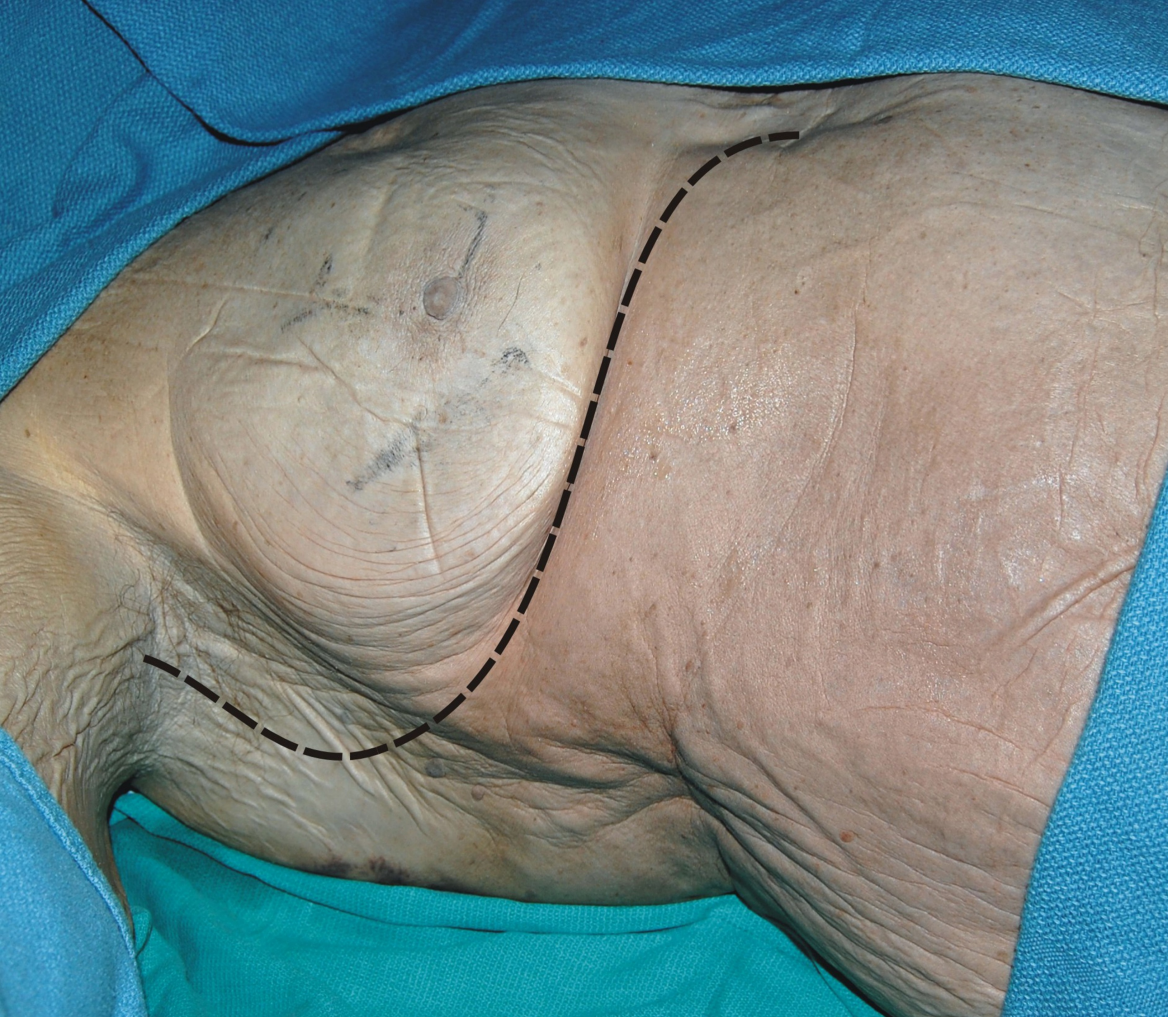
Cadaveric example of skin incision (dotted lines)

**Figure 2:**
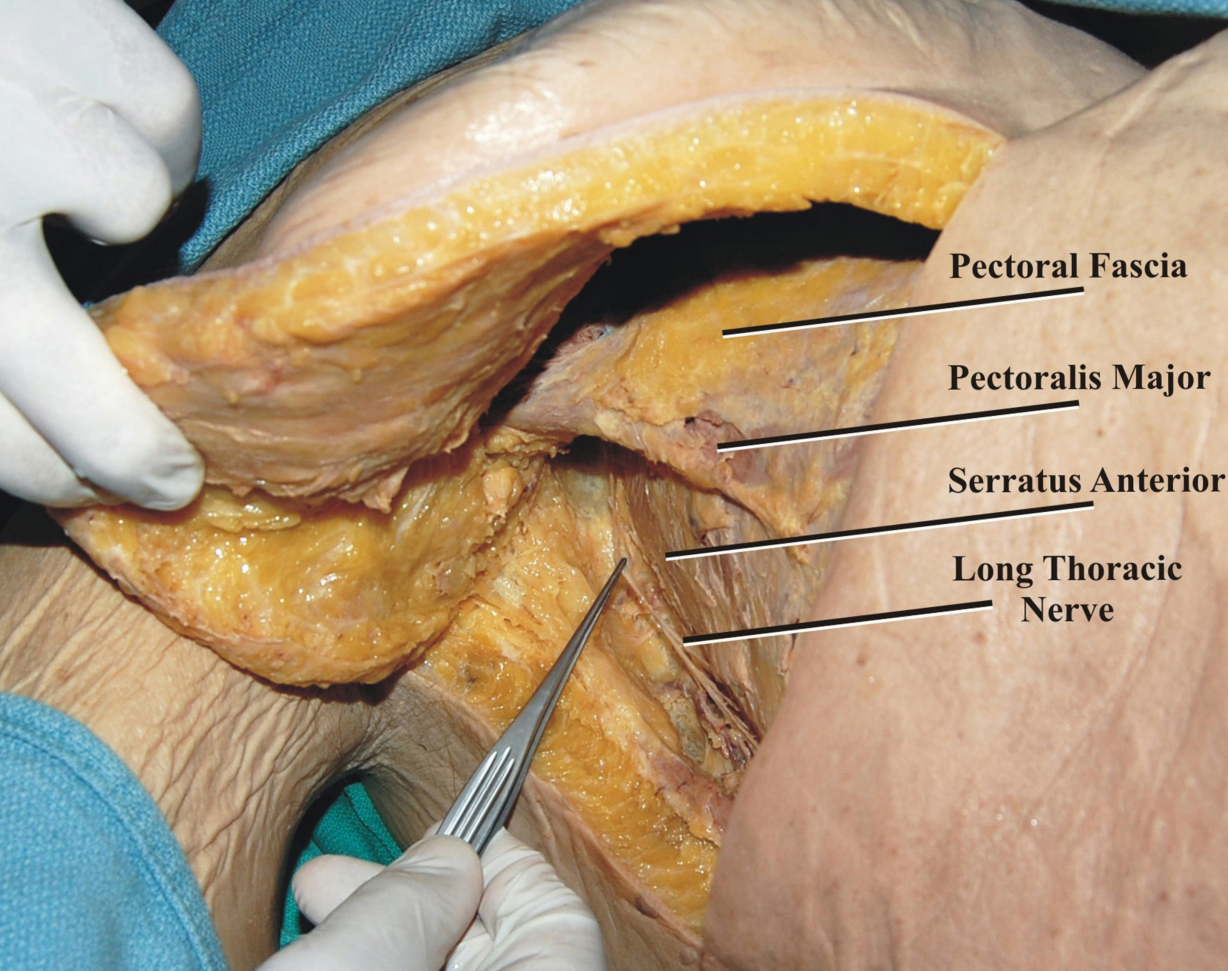
The long thoracic nerve is seen following skin retraction and superficial dissection of the lateral chest wall

**Figure 3:**
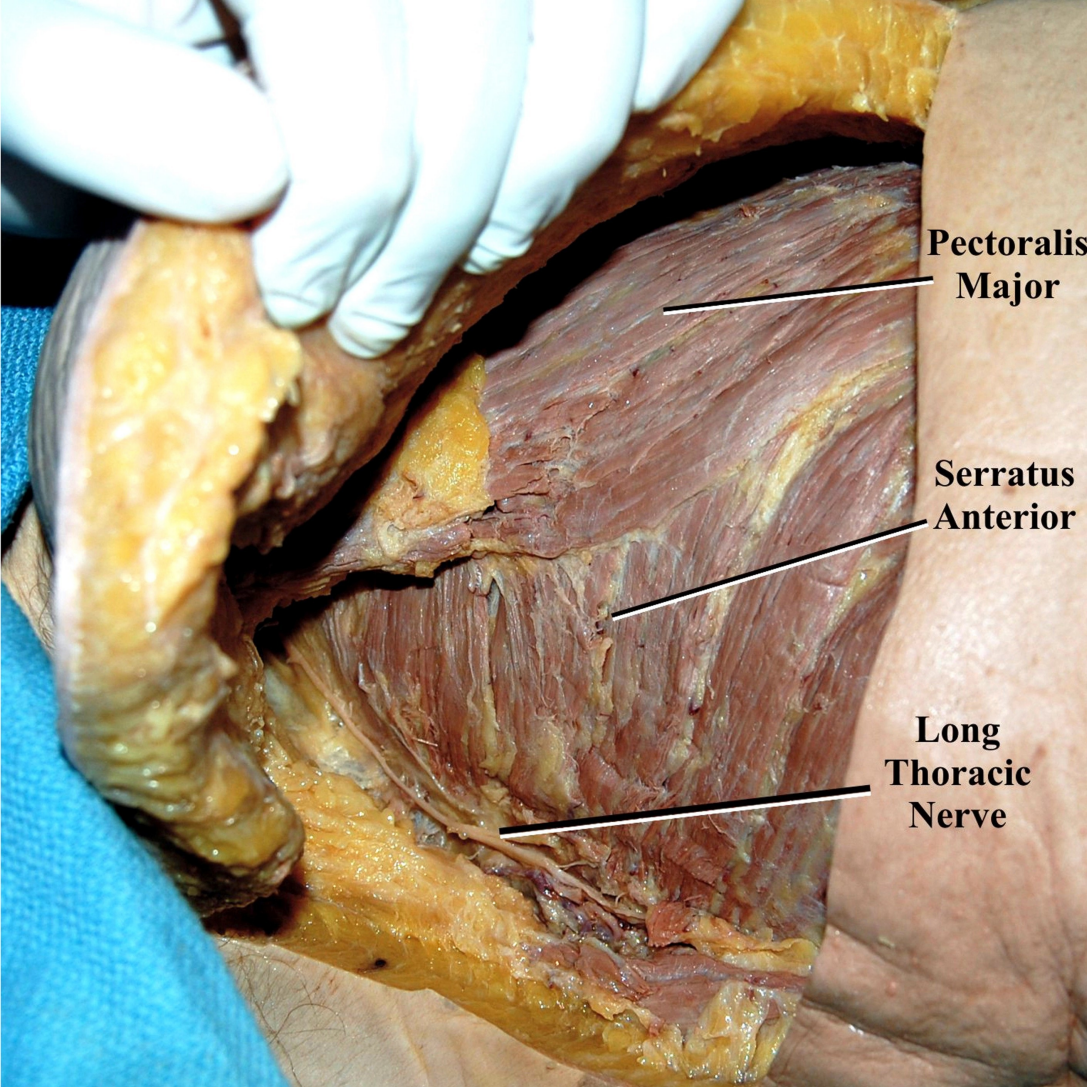
Additional dissection highlights the underlying serratus anterior muscle

**Figure 4:**
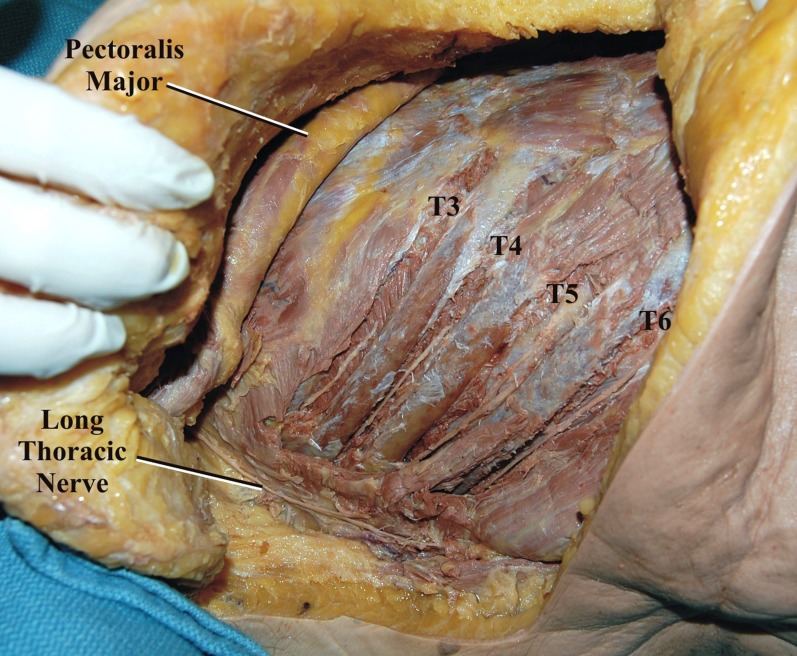
The adjacent intercostal nerves (T3-T6) are dissected in the intercostal spaces

With each cadaver in the supine position, a C-shaped incision was made, beginning in the axilla at the mid-axillary line and curving anteriorly along the fifth intercostal space toward the sternum at the midline (Figure 1). The LTN was dissected in its entirety, inferior to the edge of the pectoralis major over the lateral chest wall superficial to the serratus anterior muscle (Figures 2-3). Additionally, the adjacent ICNs in the third through sixth intercostal spaces were dissected and cut distally and mobilized to the laterally placed LTN (Figure 4). Measurements included the diameter of the LTN overlying the lateral chest wall and the diameter of the adjacent ICN. Statistical analysis was performed using the Student t-test (Statistical Package for the Social Sciences 8.0 for Windows) and significance was set at p <0.05.

 

 

## Results

**Figure 5:**
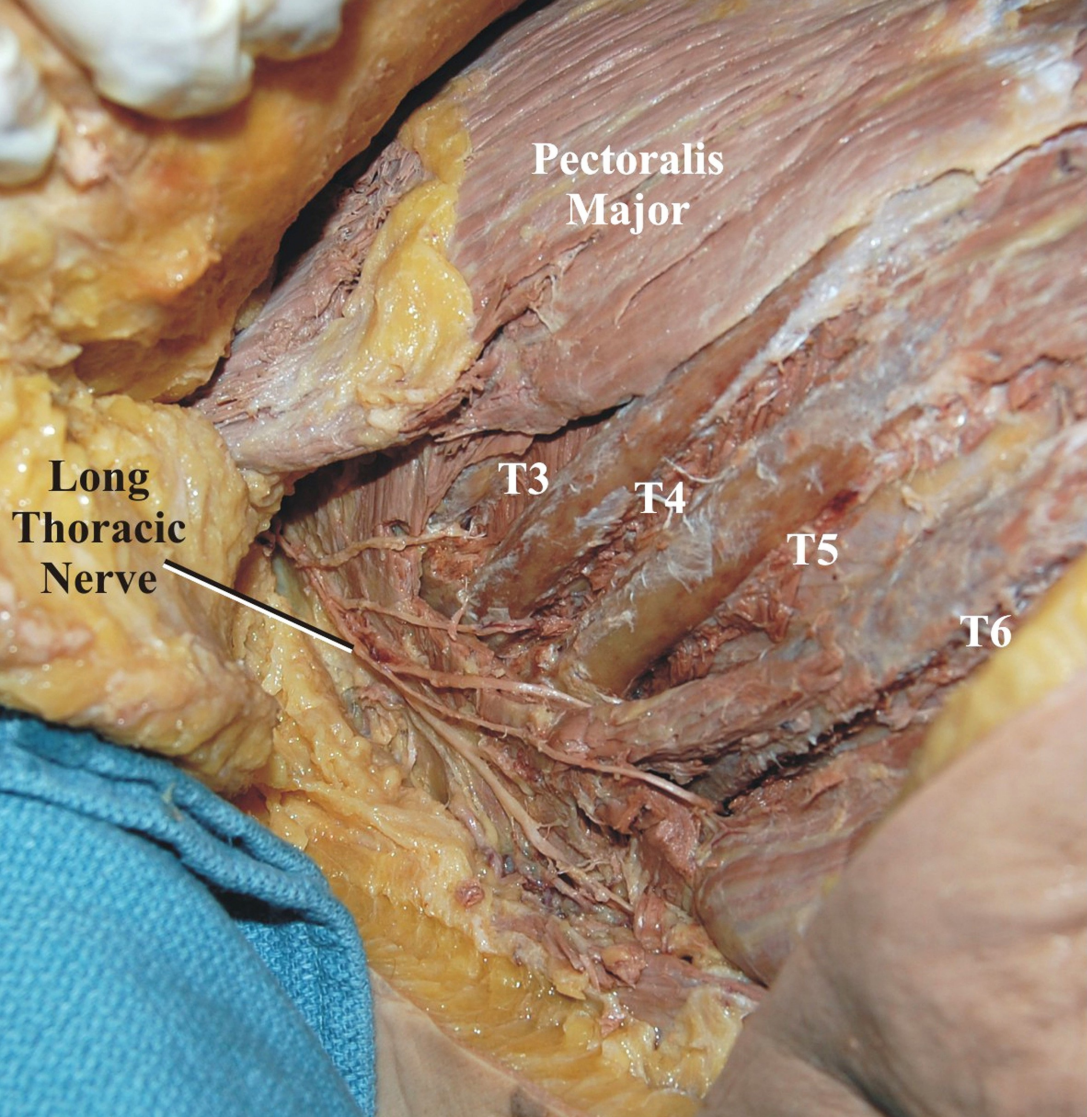
The intercostal nerves are transposed laterally to the long thoracic nerve

No abnormalities in the gross morphology of the LTN was observed in any of the specimens. The LTN was easily identified in the superficial tissues over the serratus anterior muscle and beginning at the lower edge of the pectoralis major muscle. This was felt to be the ideal site to section the LTN recipient in order to provide a maximal length for mobilization while still maintaining the adequate cross-sectional area for end-to-end anastomosis. Within the operative site, the mean proximal and distal diameters of the LTN were 1.6 mm (range 1.44 mm-1.85 mm) and 1.1 mm (range 1.0 mm-1.4 mm), respectively. The adjacent ICNs had a mean diameter of 1.3 mm (range 1.0 mm-1.5 mm). On all sides, the ICN branches were easily transposed to the adjacent LTN without any tension (Figure 5). Connection to the LTN could be achieved without tension for the third to sixth ICNs provided each nerve was dissected anteriorly to the midclavicular line. The end to end size match between donor and LTN was appropriate on all sides. No statistical differences were noted for any of the measurements comparing sides or sex.

## Discussion

**Figure 6:**
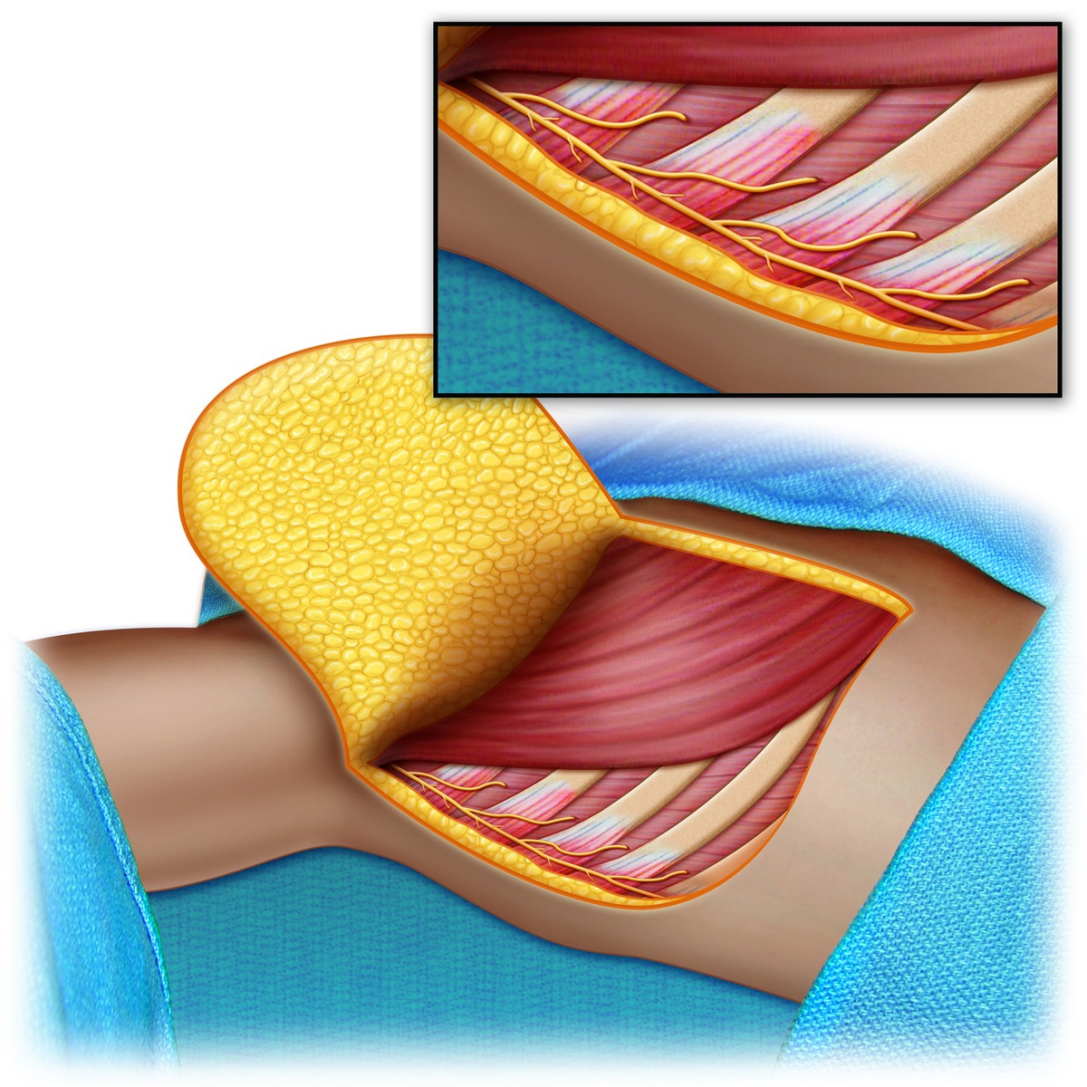
Schematic drawing of the intercostal nerve to the long thoracic nerve neurotization procedure

Based on our cadaveric study, the transposition of the adjacent ICN to the LTN over the lateral chest was easily performed (Figure 6). The size of the ICNs to the size of the LTN was appropriate. Therefore, neurotization using the ICNs in patients with LTN injury might be considered.

Injury to the LTN with a resultant winged scapula was first described by Velpau in 1837 [17]. While the clinical syndrome of winged scapula can also arise from injury to the accessory nerve, the LTN injury is more commonly the culprit. The treatment options available for winged scapula from LTN injury can be broadly divided into two categories: structural/orthopedic and functional procedures involving nerve repair or transfer. In general, the structural or orthopedic solutions involve the transfer of various shoulder girdle muscles with or without soft tissue grafts [18]. In 1904, Tubby first attempted a structural repair for the treatment of winged scapula by transferring fascicles from pectoralis major to the serratus anterior muscle, but was unsuccessful. Several authors have attempted both fascial slings and dynamic tendon transfers involving the rhomboids, pectoralis minor, teres major, levator scapulae, and pectoralis major with varied success [18-20]. Recently, Galano, et al. described the use of split pectoralis major muscle graft in a series of 10 patients with serratus anterior muscle palsy [18]. In that series, they reported positive outcomes for functional recovery based upon improvements in the American Shoulder and Elbow Surgeon Scores. It is important to recognize that of the two patients with functional limitation of shoulder elevation, only one improved from 60 to 110 degrees of elevation, while the other achieved 90 degrees of elevation both before and after the graft was performed. A complete review of the various techniques and outcomes for orthopedic repair of serratus anterior muscle palsy is beyond the scope of this paper. These orthopedic procedures would intuitively serve as second-line therapies for the treatment of a problem that is primarily neurologic. They have, thus far, taken center stage as primary nerve repair and nerve transfer procedures have been either unavailable or ineffective.

Less than 50 years ago, surgical treatment of brachial plexus injuries was considered a wasted effort yet advances in modern surgical techniques and technology have significantly improved outcomes. One of the main reasons that neurotization procedures have not been more readily available for the treatment of winged scapulae is that they are time dependent. Operating early might not allow sufficient time for spontaneous reinnervation and waiting too long can lead to failure of the motor end plate and thus failure of reinnervation [1]. Several authors have described the advantages of delayed exploration and repair for brachial plexus injuries, with the optimal time for surgery considered between three and six months after the initial injury [1, 21]. Jivan, et al. presented a series of 27 patients who underwent nerve grafting to the upper trunk to restore shoulder and biceps brachii function. They found that the best recovery of function occurred in those patients who underwent surgery within two weeks of injury, whereas those who had a delayed repair (greater than two months) had a significantly less functional recovery of biceps brachii and deltoid strength [22]. In evaluating outcomes for patients who underwent brachial plexus neurotization with ICN donors, Moiyadi, et al. found that the time of muscle denervation was the most important predictor of patient improvement and that the most improvement occurred at 5.5 months [23]. While the critical time point may vary with each data set, the trend indicates that early evaluation and intervention (within six months) by a surgeon is critical for a reasonable chance for successful neurotization. Unfortunately, in practice, this is often the limiting factor as many cases of winged scapula are delayed in their diagnosis or misdiagnosed as other entities [18].

The theoretical advantages of ICNs are that they are readily available for harvest with minimal complications and are not involved in ipsilateral brachial plexus injury. In addition, multiple donor nerves can be harvested for transfer to several injured segments of the brachial plexus. This becomes important in cases of complete brachial plexus injuries when priorities to reinnervation must be considered. Most neurosurgeons would agree that elbow flexion is the most important function to obtain, followed by debatably, shoulder abduction, external rotation, hand sensibility, wrist and finger extension and flexion and finally, intrinsic function of the hand [1]. One common combination is accessory to suprascapular nerve transfer for shoulder abduction plus intercostal to musculocutaneous nerve transfer for elbow flexion. While some insist that axillary nerve repair is crucial for shoulder reanimation, Yamada, et al. argued that biomechanically, the deltoid contributes to phases of shoulder abduction, which can all be supplemented by other shoulder girdle muscles: the first phase (0 to 90 degrees) by the supraspinatus and long head of the biceps brachii, the second phase (90 to 150 degrees) and third phase (150 to 180 degrees) by the trapezius and serratus anterior [24]. Based on our results, even in cases where the third and fourth ICNs must be necessarily prioritized for transfer to the musculocutaneous nerve, the fifth and sixth intercostals would still be available for transfer to the LTN, thus allowing an almost complete restoration of the elbow flexion and shoulder abduction.

A survey of experienced peripheral nerve surgeons revealed ICNs as second only to the accessory nerve as the preferred choice of donor for nerve transfers. Several authors have demonstrated the suitability of the ICNs for nerve transfer in brachial plexus injury [23, 25-27]. Moiyadi, et al. reported on a series of 51 patients who underwent neurotization with ICNs for brachial plexus injuries. Overall, they reported functional improvement in 58% of patients, including musculocutaneous, axillary, ulnar, and radial nerve recipients [23]. Wood and Krakauer reported that 9 of 13 patients who underwent intercostal to musculocutaneous nerve transfer regained useful elbow flexion [26]. Similarly, Nagano, et al. reported that out of 112 patients treated with intercostal to musculocutaneous nerve transfer, 87% regained grade 3 or 4 elbow flexion [27]. While neurotization with ICNs has been used successfully to restore the function of numerous brachial plexus components (musculocutaneous, axillary, radial, and ulnar), only one report exists in the literature that describes LTN neurotization with ICN donors. Yamada, et al. reported on a single case in which intercostal to LTN nerve transfer was used in combination with the accessory nerve to suprascapular nerve neurotization to achieve functional restoration of shoulder mobility including 180 degrees of active shoulder flexion and abduction with elimination of scapular winging [24]. It is crucial to emphasize the importance of identifying which injuries require LTN repair based on whether or not C7 is involved. In preganglionic injuries of C5 and C6 nerve roots, the lower part of the serratus anterior muscle remains intact to stabilize the scapulothoracic joint and prevents winging of the scapula. Conversely, if C7 is also involved, the entire serratus anterior is lost and therefore, LTN neurotization should be considered.

## Conclusions

We found that it is feasible to harvest adjacent ICNs and move them to the adjacent LTN. Such a procedure, after being confirmed in patients, might offer a new technique for restoring protraction following an LTN injury.
